# Combined central serous chorioretinopathy, hypermetropia, short axial length, chorioretinal folds, enlarged/thickened ocular coats, with varying association of scleral changes (CHAFES)

**DOI:** 10.1186/s12886-023-03038-5

**Published:** 2023-07-14

**Authors:** Susan M Downes, Sonia P Mall, Saoud Al-Khuzaei, Rasmeet Chadha, Andrew Gibson, Victor Chong, Alan C. Bird

**Affiliations:** 1 Oxford Eye Hospital, John Radcliffe Hospital, Oxford University Hospitals NHS Foundation Trust, Oxford Eye Hospital,, West Wing LG1, Headley Way, Oxfordshire, OX3 9DU England, UK; 2 Nuffield Laboratory of Ophthalmology, Nuffield Department of Clinical Neuroscience, University of Oxford, Oxford, UK; 3 Ashford and St Peter’s Hospitals NHS Foundation Trust, Ashford, UK; 4 James Cook University Hospital, South Tees Hospitals NHS Foundation Trust, Middlesbrough, UK; 5 UCL Institute of Ophthalmology, Bath Street, London, EC1V 9EL UK; 6 Moorfields Eye Hospital, City Road, London, EC1V 2PD UK

**Keywords:** Central Serous Chorioretinopathy (CSC), Chronic, Choroidal folds, Chorioretinal folds, Posterior pole flattening, Ocular coats thickening, T-Sign, Hypermetropia, Short axial length

## Abstract

**Purpose:**

To describe a condition with the following features: chronic central serous chorioretinopathy (CCSC), chorioretinal folds, scleral changes (including any of the following flattened or ‘squared off’ posterior pole, ‘T sign’, or thickened ocular coats), accompanied by a short axial length and hypermetropia in a series of 7 patients.

**Methods:**

The case notes of 7 patients presenting with a combination of CSC, choroidal folds scleral changes and hypermetropia were reviewed as part of a retrospective case series. Corrected visual acuities, serial refraction, colour imaging, fluorescein and indocyanine green angiography findings, together with B-ultrasound scan features were recorded, with axial length measurements as available (< 23.3 mm was defined as short).

**Results:**

The study included 14 eyes of 7 subjects (2 females and 5 males) with a primary presentation of central vision disturbance. All patients showed signs of previous or current episodes of the following features in at least one eye: CSC (5/7 bilateral); choroidal folds (6/7 bilateral), thickening of ocular coats in the 5 in whom this was measured, at least one scleral abnormality on ultrasound in at least one eye. A short axial length at final appointment was recorded in 13/14 eyes.

**Conclusions and relevance:**

The combination of CCSC with choroidal folds, hypermetropia with apparent shortening of the eyeball associated with one or more scleral abnormalities such as a flattened or ‘squared off ‘appearance of the B ultrasound may be a specific ocular condition. The aetiology of this particular combination of posterior segment manifestations is unknown; the choroid could be the primary focus of disease with secondary involvement of the sclera. Alternatively, the features observed may result from a chronic inflammatory process affecting the sclera with secondary effects on the choroid, retinal pigment epithelium and retina. In our case series, the final vision was not significantly different from vision at presentation.

## Introduction

The coexistence of choroidal or chorioretinal folds (CRF), and choroidal thickening, a flattened posterior pole in the context of short axial lengths and hyperopic eyes has been described previously [1–5]. The combination of CRF, and central serous chorioretinopathy (CSC) in the context of acquired hypermetropia, and a short axial length has also been reported [1, 2, 5–11]. However, to our knowledge the combination of all these features has not been previously reported.

In this case series of 7 patients, we describe eyes in which both CSC and CRF are present; with short axial lengths and scleral changes (flattening, ocular coats thickening and/or a ‘T’ sign). Some or all of these features were seen at presentation or developed during follow up. We also report increasing hypermetropia and shortening axial lengths in a proportion of our patients.

Choroidal folds (CF) are not common and were first described by Nettleship in 1884 [3]. Gass proposed that all cases of CF with a hyperopic shift were likely to be acquired [5]. CF have been described in association with a large range of ocular disorders including hypermetropia, macular degeneration, scleral buckling surgery, hypotony post cataract or glaucoma surgery, optic neuropathy, trauma, orbital tumours, thyroid eye disease, scleritis and papilloedema. They are also seen in the context of systemic diseases such as intracranial hypertension, infections, neoplasia, and autoimmune conditions [6]. The co-existence of CF and flattening of the posterior ocular wall with acquired hypermetropia was described by Dailey et al. [2]. In 1979 Kalina and Mills reported CF and acquired hypermetropia in six healthy adults, three of whom had flattening of their posterior pole on B-ultrasonography [1, 12]. Stimak et al. describe seven patients with a diagnosis of acquired hypermetropia and CF where flattening of the globe was seen and investigated their patients with CT imaging, which showed variable enlargement of the optic nerve complex [4].

CSC is a condition characterised by serous detachment of the neurosensory retina and retinal pigment epithelium and tends to affect males more than females [13] peaking at ages 40–50 years. Chronic CSC is characterised by retinal pigment epithelial damage leading to atrophy in some cases with ensuing visual impairment [13]. Cohen et al. described 6 patients with the combination of CSC and CRF [8]; and this has been described in other case reports [10, 14]. Imamura et al. identified 5 patients with CF in 238 patients with CSC [15].

We present here a series of 7 patients with the combined features of CRF, CSC, with increasing hypermetropia and thickening of the posterior pole with associated progressive shortening of the eyeball, with thickening of the posterior pole with relatively preserved vision.

## Materials and methods

The clinical notes of 7 patients seen at the Oxford Eye Hospital and identified with a combination of CSC, and CRF were then investigated for any scleral changes/ocular coat thickening together with axial length and refraction measurements. Clinical data included the patients’ medical history, risk factors for CSC and examination and investigations. These included best corrected visual acuity, serial refraction where available, slit-lamp biomicroscopy and fundoscopy, digital colour fundus photography, short-wavelength (488) fundus autofluorescence (Spectralis; Heidelberg Engineering, Heidelberg, Germany), spectral domain–optical coherence tomography (SD–OCT; Spectralis, Heidelberg Engineering, Heidelberg, Germany). Ultrasound B scan axial lengths were measured and features including flattening of the posterior pole, the presence of a T-sign and thickness of the ocular coats were recorded. The axial lengths were measured by putting callipers on the B scan image to measure the length from the anterior corneal surface to the internal limiting membrane. The axial length was an approximate measurement because they were measured using a B scan.

Patients were consented for publication of their anonymised imaging and medical data.

## Results

The study included 14 eyes of 7 subjects (2 females and 5 males) age at presentation ranged from 45 to 76 with a primary presentation of central vision disturbance. All patients showed signs of previous or current episodes of the following features in at least one eye: CSC (5/7 bilateral); choroidal/chorioretinal folds (6/7 bilateral), thickening of ocular coats in the 5 in whom this was measured, at least one scleral abnormality on ultrasound in at least one eye. The Clinical details of patients with CRF and CSC are summarised in Table 1.

**Table 1:** Clinical details of patients with choroidal/chorioretinal folds (CRF) and central serous chorioretinopathy (CSC)

Patients (sex)	1 (M)	2(F)	3 (M)	4 (M)	5(F)	6 (M)	7 (M)
Age	47	59	75	45	76	63	56
Symptom of ↓vision	OS	OS	OU	OD	OS	OU	OS
Years of FU	13	10	5	6	7	5	< 1
CSC present	OU	OS	OU	OU	OS	OU	OU
Choroidal folds present	OU	OU	OD	OU	OU	OU	OU
Ultrasound anomalies							
Flattened posterior pole	OU	OU	Insufficient imaging quality	OU	OU	OU	Normal
Ultra sound T sign	No	No	NP	No	OU	No	No
Thickened ocular coats	NP	OS	OU	NP	OU	OU	OU
OD	NP	1.60 mm	2.42 mm	NP	2.27 mm	2.06 mm	1.84 mm
OS	NP	2.52 mm	2.31 mm	NP	2.08 mm	2.12 mm	1.86 mm
Ocular co-morbidities	Minimal epiretinal changes	Nil	Choroidal polyps/PDT	Nil	Nil	Nil	Nil
Systemic Co-morbidities	Nil	Nil	Rheumatoid arthritis on steroids, HCQ	Nephrotic syndrome on steroids	Choroidal naevi – flat	Treated ↑BP	Treated ↑BP, steroid injection (frozen shoulder)
TFTs	n	n	NP	NP	n	NP	NP
MRI/CT	n	n	NP	NP	n	NP	NP

BP: blood pressure, CNV: choroidal neovascularisation, F: flattened, HCQ: hydroxychloroquine, OD: right eye, OS: left eye, OU: both eyes, n: normal, NP: not performed, PDT: photodynamic therapy

The refraction ranged from − 3.0 to + 8.0 (see Table 2). Serial refraction was available for 4 patients. At baseline 10/14 eyes were hypermetropic, and 4/14 eyes were myopic follow up was available for 8 eyes; of these 6 eyes were hypermetropic and 2 showed less myopia than at baseline (Patient 2). One eye of one patient was noted to be slightly less hypermetropic (0.5 dioptres) at their final visit (patient number 2).

Axial lengths ranged from 20.33 to 24.38 mm at baseline visit in 14 eyes and 20.74 to 23.10 mm follow up in the four eyes that had follow up data (normal mean average axial length reported in the literature is 23.3 mm [16]) (see Table 2). Two of the 7 patients (4 eyes) had serial axial length measurements (non-immersive ultrasound scans) of at least 2 years of follow up. At presentation 9/14 eyes had a short axial length, and by final appointment a short axial length was recorded in 13/14 eyes.

**Table 2:** Changes in visual function and axial lengths over the follow up period in patients with chorioretinal folds (CRF) and central serous chorioretinopathy (CSC)

		VA start	VA finish	Refraction Start	Refraction finish	AL a presentation	AL Final visit	Axial length change + = longer eye at end - = shorter eye at end	Refraction change + = hyperopic shift - = myopic shift
1	OD	6/4	6/6	-3.00	-0.50/-0.50 × 95 (BVS − 0.75)	24.38	22.85	-1.53 mm	+ 2.25 DS
	OS	6/9	6/4	-0.75/-0.25 × 180 (BVS − 0.875)	-0.25/-0.25 × 150 (BVS − 0.375)	23.67	21.74	-1.93 mm	+ 0.50 DS
2	OD	6/6	6/7.5	+ 3.25/ -2.50 × 165 (BVS + 2.00)	+ 3.75/-2.50 × 166 (BVS + 2.50)	22.29	21.86	-0.43	+ 0.50 DS
	3OS	6/7.5	6/12	+ 3.50/-1.75 × 10 (BVS + 2.625)	+ 3.00/-1.75 × 7 (BVS + 2.125)	21.94	21.42	-0.52 mm	-0.50 DS
3	OD	6/18	6/30	+ 7.00/ +2.00 × 95	+ 7.00/ +2.00 × 95	20.33	NP	NP	0.00 DS
	OS	6/120	6/60	+ 8.00/ +1.00 × 80	+ 8.00/ +1.00 × 80 4,621,979	20.44	NP	NP	0.00 DS
4	OD	6/36	6/18	-1.00/-0.75 × 10 (BVS − 1.375DS)	NP	22.51	NP	NP	NP
	OS	6/5	6/9	-2.75/-0.75 × 170 (BVS − 3.125DS)	NP	24.06	NP	NP	NP
5	OD	6/6	6/18	+ 4.00	+ 4.00	NP	21.25	NP	No change
	OS	6/6	6/6	+ 3.75	+ 3.75/ -1.25 × 100 (BVS + 3.125)	NP	21.35	NP	-0.50 DS
6	7 OD	6/6	6/7.5	NP	+ 2.00/-2.00 × 97 (BVS + 3.00)	21.96	NP	NP	NP
	7 OS	6/15	6/6	NP	+ 1.75/-1.25 × 82 (BVS + 2.50)	22.87	NP	NP	NP
7	OD	6/6	6/6	NP	+ 3.50/-0.50 × 30 (ad + 2.25)	21.86	NP	NP	NP
	OS	6/6	6/9	NP	+ 5.00/-0.50 × 70 (add + 2.25)	21.13	NP	NP	NP

NP: not performed

**Fig. 1:**
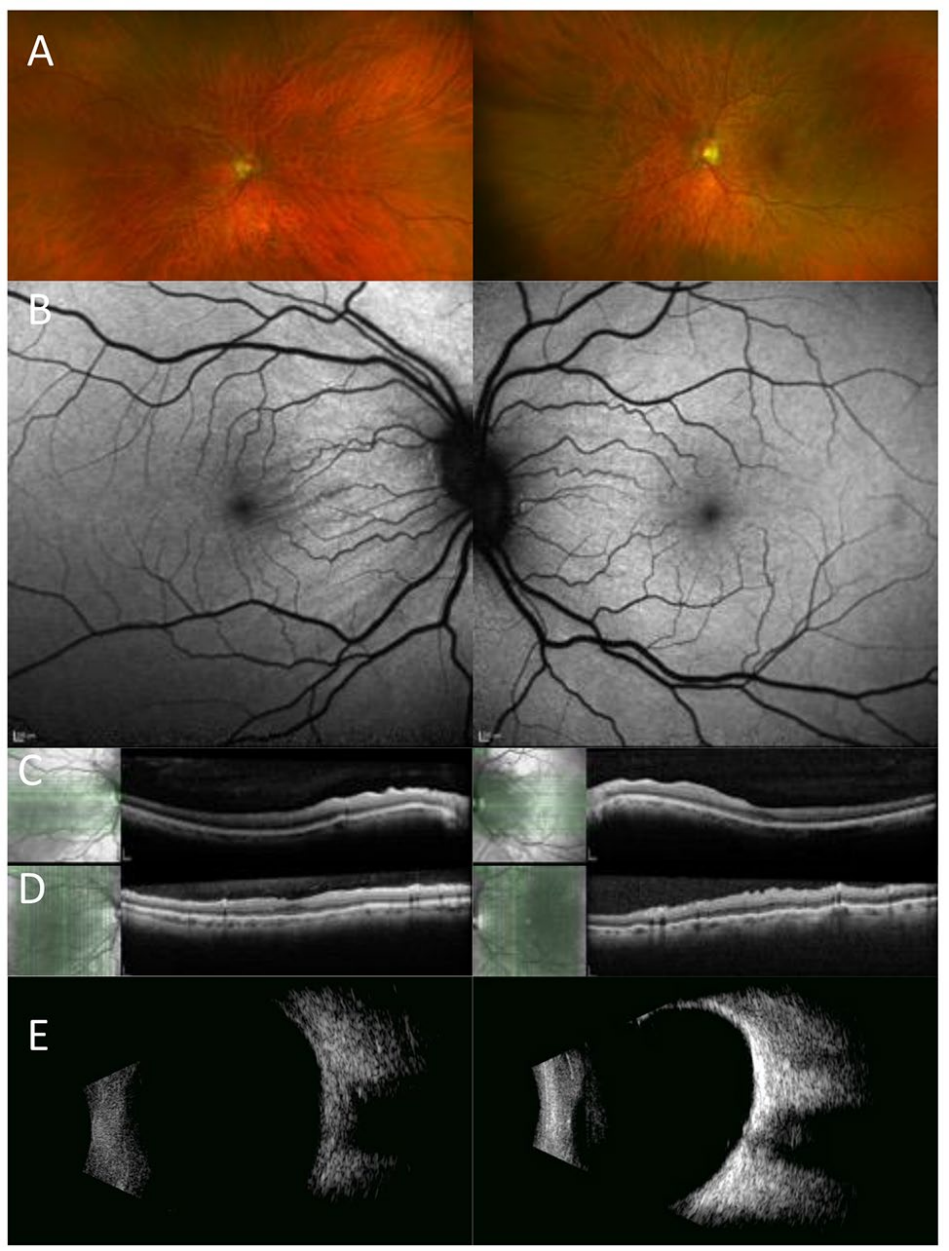
Multimodal imaging of case 1 **A**) Optos imaging showing chorioretinal folds in the macular region **B**) CRF seen more clearly on the AF imaging in both eyes **C**-**D**) OCT imaging at baseline (c) and follow up (**D**) showing bilateral chorioretinal folds **E**) B scan showing bilateral flattened posterior poles

**Fig. 2:**
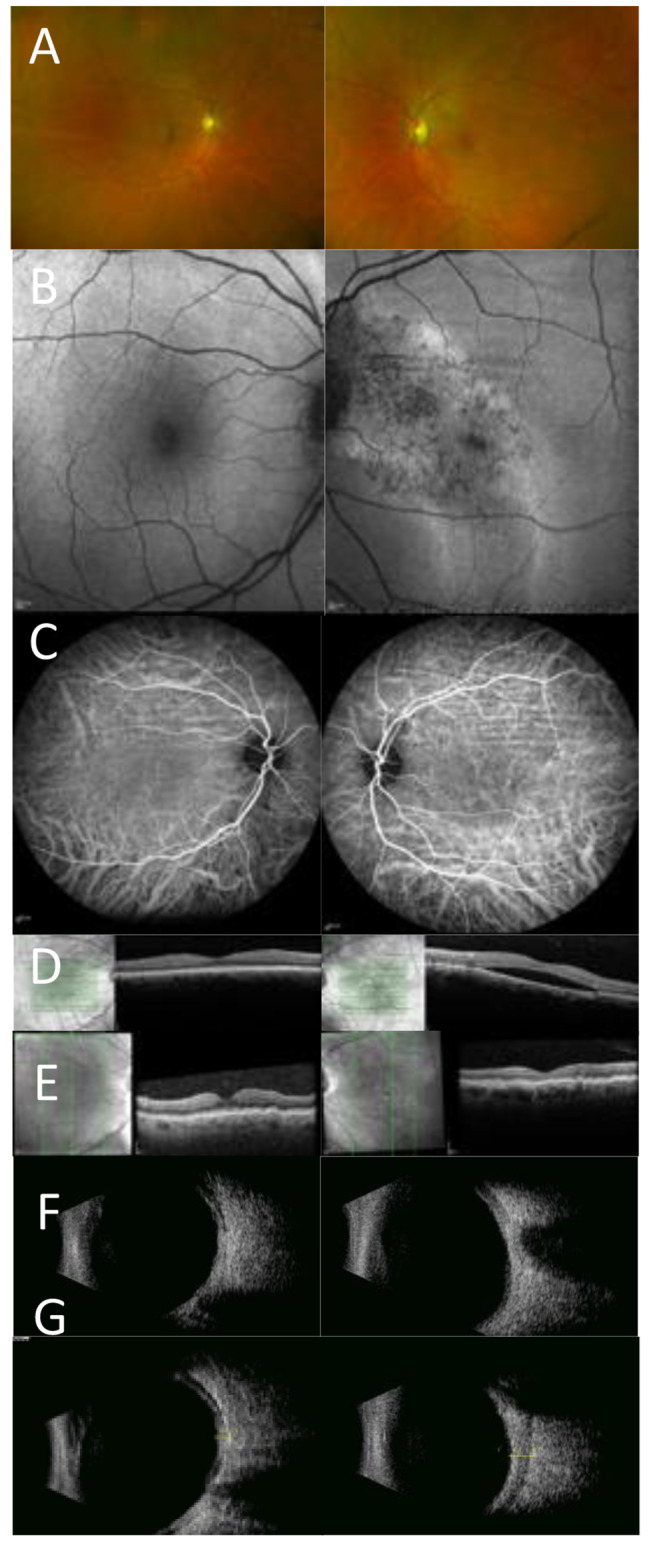
Multi-modal imaging for case 2. **A**) Optos imaging showing CRF in the macular region; more pronounced in the left eye **B**) FAF imaging showing features of chronic CSC with gravitational tract in the left eye, and CRF that are more pronounced in the left eye (**C**) ICGA imaging showing bilateral CRF (**D**-**E**) OCT imaging using horizontal scan at baseline(**D**) showing serous fluid in the central macula of the left eye and follow up OCT vertical scan (**E**) showing bilateral chorioretinal folds with resolution of the CSC neurosensory retinal elevation (**F**) Ultrasound B scan showing bilateral flattened posterior poles. (**G**) and thickened ocular coats in the left eye

**Fig. 3:**
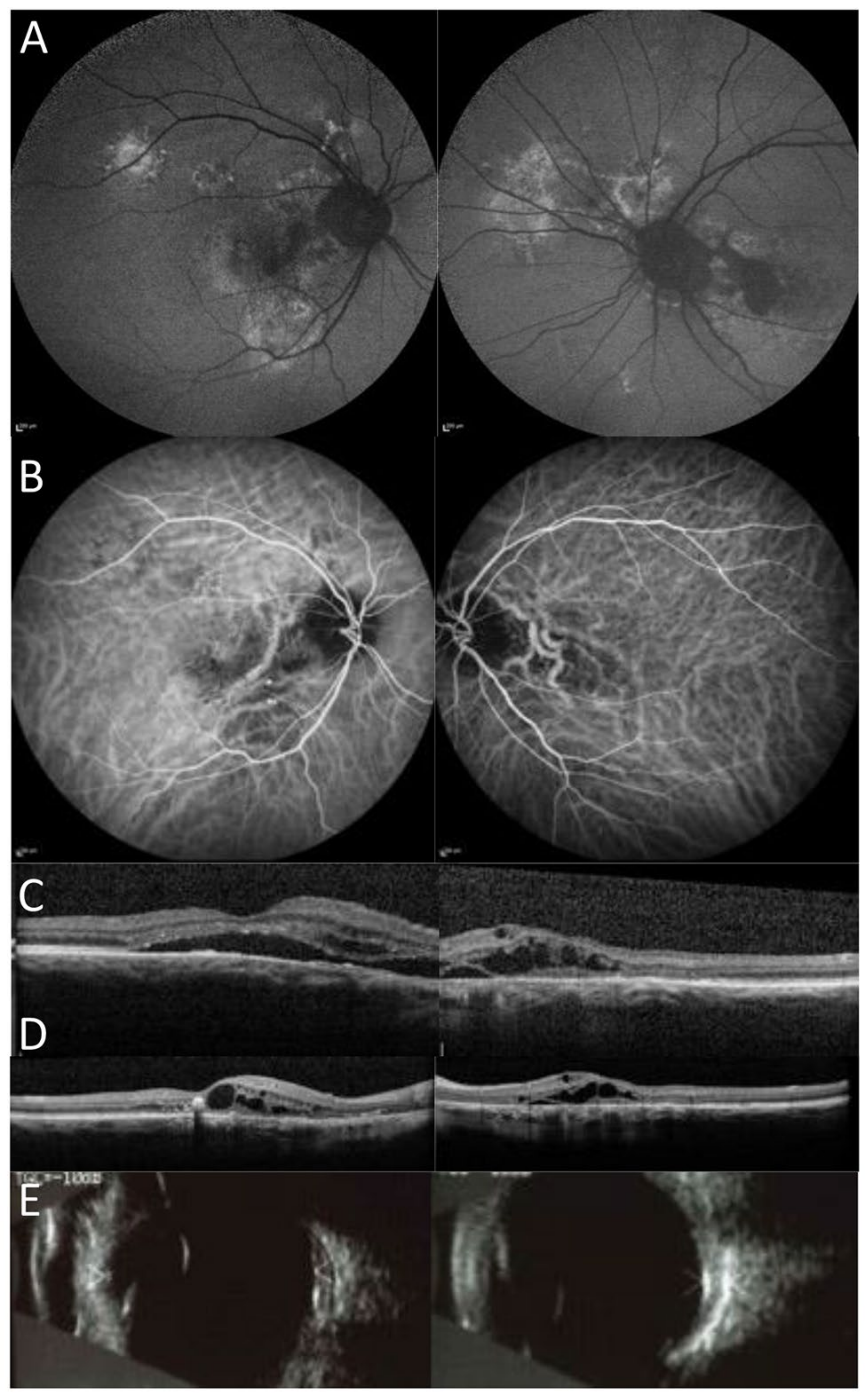
Multi-modal imaging for case 3 **A**) Fundus autofluorescence (FAF) imaging showing features of current and previous episodes of CSC in both eyes (**B**) ICGA imaging demonstrating CRF in the right eye and polypoidal choroidopathy (**C**-**D**) OCT imaging at baseline (**C**) showing bilateral CSC and intraretinal fluid and follow up OCT imaging (**D**) improvement of the CSC but with remaining intra-retinal fluid (**E**) Ultrasound B scan images showing bilateral thickened ocular coats.

**Fig. 4:**
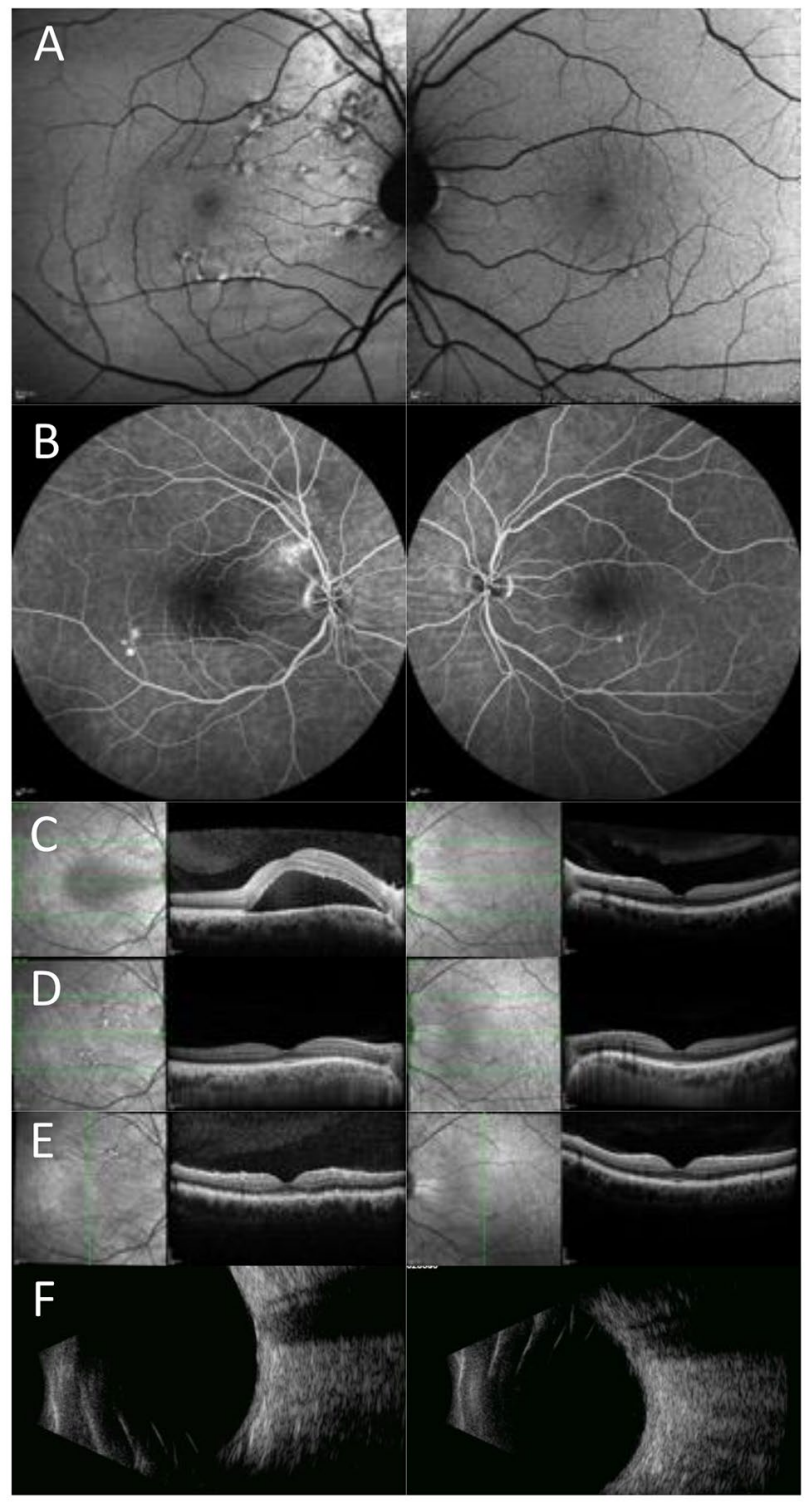
Multi-modal imaging for case 4 **A**) Fundus autofluorescence (FAF) imaging showing features of active focal CSC in the right eye and features of a previous CSC in both eyes (**B**) FFA imaging demonstrating CRF in macular region of the right eye and areas of hyperfluorescence consistent with chronic CSC. The left eye has CRF nasal to the optic disc and a hyperfluorescent spot consistent with CSC. (**C**-**E**) OCT imaging at baseline (**C**) shows typical neurosensory elevation of CSC in the right eye and follow up OCT imaging (**D**) shows resolution of the CSC in the right eye (**E**) OCT imaging with vertical scan shows the CRF in the macula of the right eye. (**F**) Ultrasound B scan images showing bilateral flattened posterior poles

**Fig. 5:**
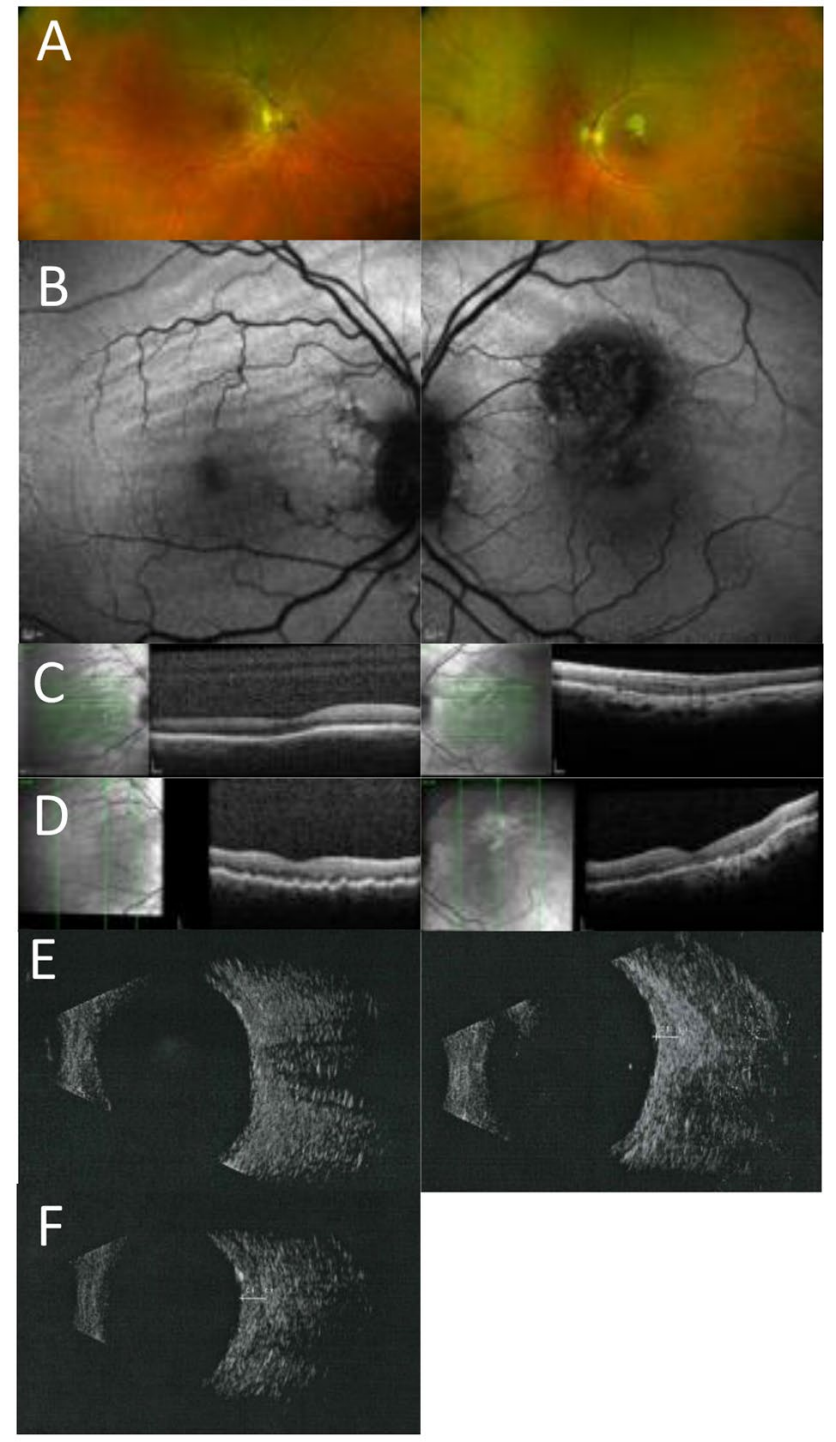
Multi-modal imaging for case 5. (**A**) Optos imaging showing bilateral CRF in the macular region, and in the left macula chorioretinal scarring and a supero-temporal choroidal naevus in the right eye (**B**) FAF imaging showing features of chronic CSC in the left eye with a reduced AF signal in the area of the chorioretinal scarring and features consistent with previous episodes of CSC in the right eye and the presence of bilateral CRF. (**C**-**D**) OCT imaging at baseline bilateral CRFs that are more obvious on the vertical scans (**E**) Ultrasound B scan showing bilateral flattened posterior poles and the T-Sign in the left eye (F) Thickened ocular coats and T sign in the right eye

**Fig. 6:**
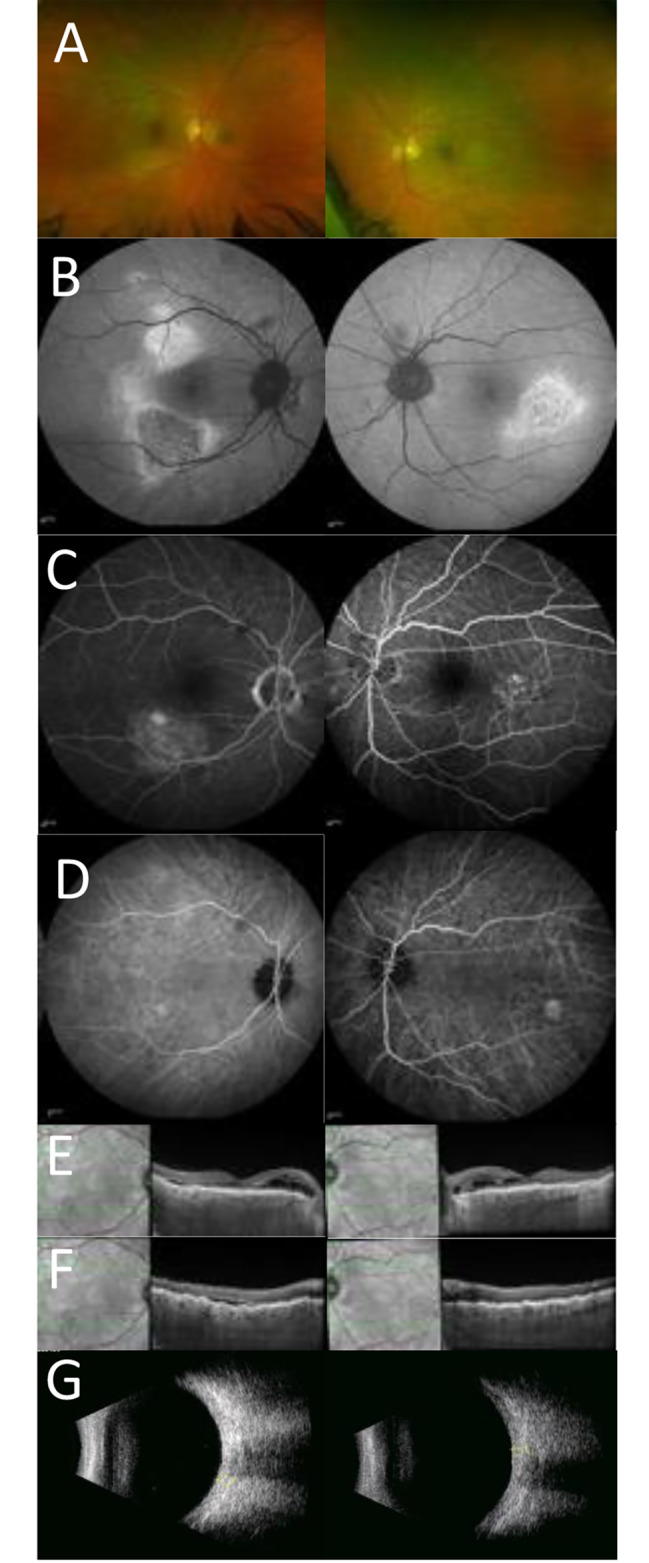
Multi-modal imaging for case 6. (**A**) Optos imaging showing areas of patchy retinal pigment epithelial pallor corresponding to areas of increased signal on FAF seen on (**B**: FAF imaging showing features of chronic CSC in both eyes and bilateral CRF. (**C**) FFA shows hyperfluorescent punctate changes in the left temporal region and staining in the right inferior macular region (**D**) ICGA ICG showed small areas of vasopermeability bilaterally and bilateral choroidal folds (**E**) OCT images showing bilateral cystic fluid in the peripapillary region (**F**) OCT **i**mages showing bilateral CRF (G) Ultrasound B scan showing bilateral thickened ocular coats and left posterior pole flattening.

**Fig. 7:**
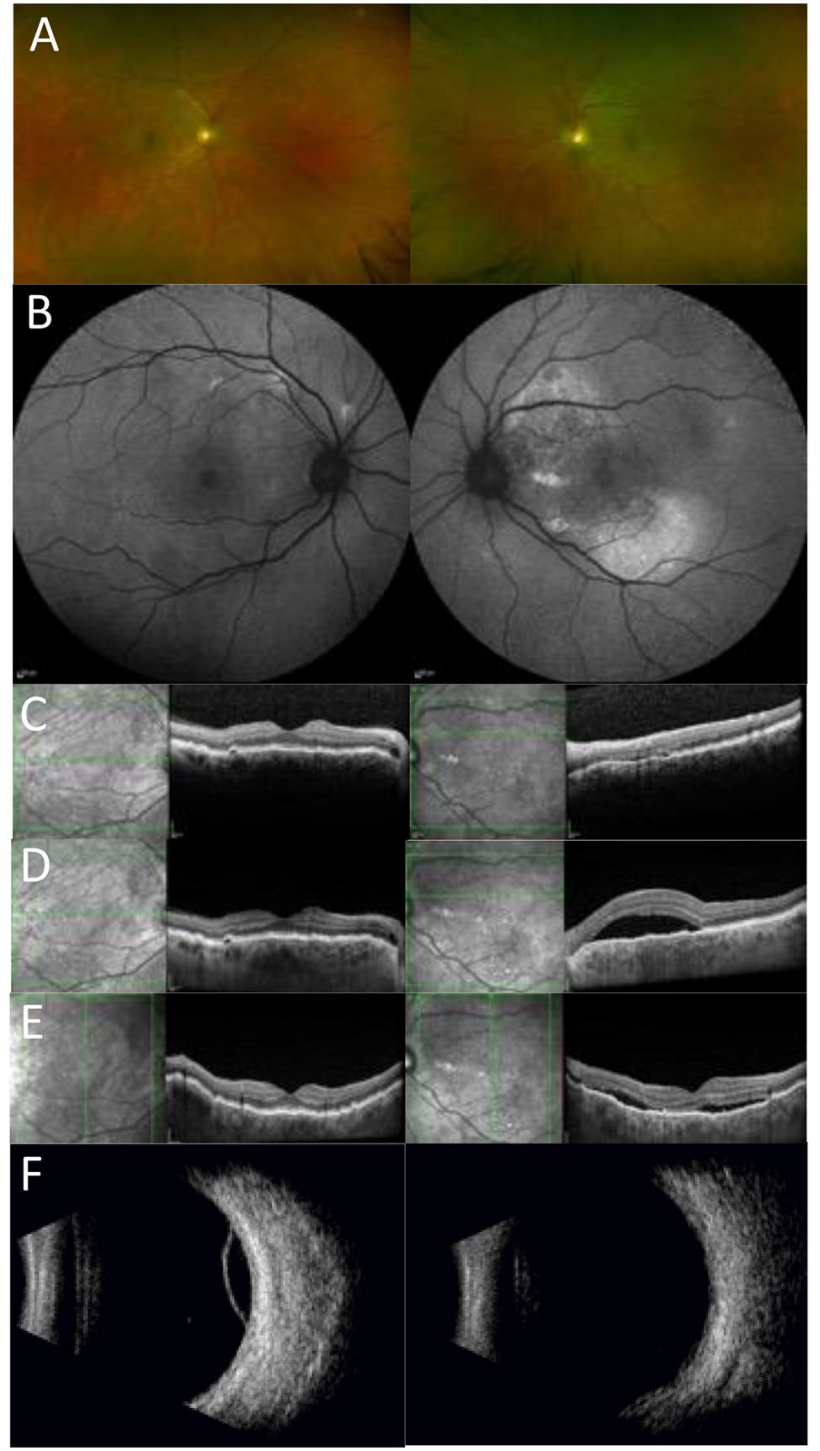
Multi-modal imaging for case 7. (**A**) Optos imaging showing CRF right eye more distinctly (**B**) FAF imaging shows bilateral raised autofluorescence signal with significant patches of AF signal increase in the left posterior pole (superior and inferior) with a decreased signal at the left fovea. (**C**) Baseline OCT images show bilateral CRF, CSC in the left eye, and a small CSC related PED in the right eye with minimal cystic fluid at the right peripapillary region (**D**) Follow up OCT images again showing the bilateral choroidal folds, significant increase of the CSC neurosensory detachment in the left eye, and stable appearance of the PED in the right eye (**E**) OCT images with vertical scan showing bilateral choroidal folds and CSC in the left eye at the same time point as in D. (**F**) Ultrasound B scan showing bilateral thickened ocular coats and peripheral retinoschisis which is more prominent in the right eye

Patient 5 had a ‘T’ sign on B ultrasound bilaterally, and 5 of 6 patients with imaging of sufficient quality were found to have flattening of the posterior pole bilaterally, and ocular coats were thickened in 9/10 eyes measured. (see Figs. 1–7).

Other ocular comorbidities were identified in two patients: patient 5 had a choroidal naevus and patient 3 who had choroidal polypoidal vasculopathy, for which they received photodynamic therapy. Systemic co-morbidities were present in 4 patients. The systemic disorders seen in our series included hypertension in 2 individuals, rheumatoid arthritis in one [1] and nephrotic syndrome in another.[1] Medications of relevance included 3 patients with a history of steroid use and one of these was also taking hydroxychloroquine. Thyroid function tests were available for 3 patients, which were all normal, and MRI/CT imaging was available for 3 patients and reported as normal.

### Case 1

A 47 year old white Caucasian male was referred due to reduced central vision and a hypermetropic shift in his left eye which had settled by the time of his review at the eye hospital. However, CRF were identified in the left eye with normal vision. Retinal imaging and fundoscopy showed features consistent with a previous episode of CSC in the right eye (Fig. 1). Repeat refraction showed bilateral reduction in myopia over a 13 year period ( Table 2). B Scan measurements showed a reduction in the axial length over a 10 year period for which measurements were available (Table 2), and a bilateral flattened appearance to the posterior aspect of the globe was observed on ultrasonography. There was no progression or activity of the CSC and visual acuity remained excellent at 6/4 in the affected eye. Medical history was negative for any systemic disorders; thyroid function tests and an MRI of the orbit were reported as showing a mildly prominent optic sheath CSF signal, but no other abnormality.

### Case 2

A 59 year old female white Caucasian presented with a sudden reduction of vision in the left eye due to CSC. CRF were present bilaterally being more pronounced in the left eye (see Fig. 2). At first examination the visual acuities were 6/6 right and 6/7.5 left. Over the 10 year review period the left visual acuity fluctuated reflecting resolution and reaccumulation of CSC associated fluid, but was 6/12 at final review (See Fig. 2). Evolution of the CSC over time resulted in foveal sparing atrophy consistent with chronic left CSC (see AF image Fig. 2). Repeat refraction showed increasing hypermetropia in the right eye, but the left eye showed a reduction of 0.5 dioptres of hypermetropia in the primarily affected left eye at final review (see Table 2). Ultrasound B scan showed a bilateral ‘squared off’ appearance of the globe, flattened posterior poles, and thickened ocular coats in the left eye. Axial length measurements of the left eye showed a short axial length which further shortened over time. Measurements of the ocular coats on ultrasound were 1.60 mm OD and 2.52 mm OS. Medical history was negative for any systemic disorders; thyroid function tests and CT of the orbit were normal.

### Case 3

A 75 year old white Caucasian male patient presented with decreased vision in his right eye and the left was also affected. Imaging and fundoscopy revealed bilateral CSC with chronic features of CSC in both eyes. CRF were present in the right eye only (see Fig. 3). The right eye developed polypoidal choroidopathy for which he received a course of anti-VEGF intra-vitreal injections and photodynamic therapy. At presentation, visual acuities were 6/18 right and 6/120 left. Repeat refraction showed bilateral stable significant hypermetropia (see Table 2). Ultrasound B scan showed bilaterally thickened ocular coats which were 2.42 mm OD and 2.31 mm OS. Both eyes had short axial lengths. Medical history was significant for rheumatoid arthritis for which he took hydroxychloroquine and prednisolone.

### Case 4

A 45 year old male Brazilian patient presented with a decrease in right central vision. Imaging and fundoscopy revealed active CSC in the right eye which resolved, with features consistent with previous episodes of CSC in both eyes and the presence of bilateral CRF (see Fig. 4). At presentation the visual acuities were 6/36 right and 6/5 left and were 6/18 right and 6/9 at follow up. Refraction at presentation showed myopic astigmatism and no follow up refraction was available. Ultrasound scan showed a ‘squared off’ appearance of the globe, flattened posterior poles (see Fig. 4) and axial length measurements showed short axial length in the right eye and within normal limits in the left eye (see Table 2). Medical history was significant for nephrotic syndrome treated with prednisolone.

### Case 5

A 76 year old white Caucasian female presented with symptoms of distortion affecting her left eye. Imaging and fundoscopy revealed chronic CSC in the left eye with features consistent with previous episodes of CSC in the right eye. Bilateral CRF and a right supero-temporal choroidal naevus are also seen (Fig. 5). At presentation visual acuities were recorded at 6/6 right and left. Repeat refraction showed unchanged right hypermetropia and a minimal reduction in the left hypermetropia with astigmatism (Table 2). Ultrasound scanning showed a flattened appearance to the globes, more pronounced in the left more than right, bilateral T-sign, and diffusely thickened ocular coats. Ocular coat thicknesses were 2.27 mm OD and 2.08 mm OS. The axial lengths were short in both eyes. Medical history was negative for any systemic disorders; thyroid function tests and CT of the orbit were normal.

### Case 6

A 63 year old white Caucasian male presented with visual distortion and difficulty with reading, and was referred by his Optometrist with a presumed diagnosis of bilateral epiretinal membranes. At the time, his visual acuities improved with a new spectacle prescription but the distorted vision persisted. Visual acuities were 6/6 right and 6/15 left at presentation improving to 6/7.5 right and 6/6 left at follow up 5 years. Refraction was only available for his most recent follow up appointment and showed bilateral hypermetropia. He had bilateral early cataracts. Imaging and fundoscopy revealed active multifocal CSC located right supero- and infero-temporally, primarily within the arcades, and left temporally (Fig. 6). Cystic fluid was located at both peripapillary regions as well as within the right superior arcade. FFA showed hyperfluorescent punctate changes in the left temporal region and staining in the right inferior macular region and ICG showed small areas of vasopermeability bilaterally. Ultrasound scan showed short axial lengths in both eyes, and thickened ocular coats (Fig. 6) which measured 2.06 mm OD and 2.12 mm OS with left flattening of the posterior pole. Medical history included high BMI, hypertension and benign prostatic hyperplasia. He also reported having significant amount of stress at the time of review.

### Case 7

A 56 year old white Caucasian male was referred by his Optometrist following detection of retinal changes on a routine ophthalmic examination. Imaging and fundoscopy showed bilateral choroidal folds, pachychoroidal features, small CSC associated PEDs in the left eye and at follow up 2 CSC lesions in the right eye, one located nasally and the other temporal to the macular region (Fig. 7). At presentation, his best corrected visual acuities were 6/6 in both eyes. At the last review 8 months later, the left had deteriorated to 6/9. He first required spectacles in his late 30’s for hypermetropia which has increased over time (see Table 2). Anterior examination was unremarkable. Ultrasound scan showed thickened ocular coats which measured 1.84 mm OD and 1.86 mm OS, peripheral retinoschisis (less prominent in the left eye), and short axial lengths which measured 21.86 mm OD and 21.13 mm OS. Medical history included hypertension and a steroid injection for a frozen shoulder 1 week before his follow up appointment.

## Discussion

We describe the co-existence of CSC and CRF, in short hypermetropic eyes in association with thickened ocular coats and the presence of scleral features of flattening, or a ‘T’ sign in at least one eye in 5/7. None of the 7 patients described here have underlying systemic disorders previously reported in association with CF. It is possible that the steroid use contributed to the development of CSC, but it is not clear how this would play a role in development of CF or scleral changes.

All 7 patients had CSC and CRF on presentation in at least one eye with a trend towards increasing hypermetropisation and shortening axial lengths. Abnormally thick coats of the eye, flattening of the posterior pole have not been previously reported as coexisting. The underlying pathophysiological mechanism for this combination of ocular features is not clear. Moon et al. described shorter axial lengths in patients with CSC not only in the affected eye, but in the other eye compared to ‘normal’ eyes in a review of 35 CSC patients [17], and our findings are in keeping with this. Stimac et al. looked in detail at the optic nerve in patients with acquired hypermetropia and CF and identified mild to moderate optic nerve enlargement and a space between sheath and the nerve [4]. This was only identified in patient 2 of the 3 patients who had CT imaging in our study. It is possible that their 7 patients in their report may have a different condition, as although they report flattening of the posterior poles, none had uveoscleral thickening or CSC. Sibony et al. describe folds in the context of papilloedema and conclude that they represent biomechanical signs of stress on the optic nerve head and associated load bearing structures [18]. Friberg proposed that CF develop due to a stress-strain type of relationship between the sclera and choroid with a reduction in the area of the inner lining of the sclera resulting in a buckling force affecting the choroid from either sclera thickening or shrinkage [19]. He also proposed that this would predispose the RPE and Bruch membrane to damage, and that this would explain the angiographic appearance he likened to angioid streaks [19]. This biomechanical mechanism may explain the reason why the sclera in our patients appears to be flattened or ‘squared off’ and even thickened, and may suggest a mechanism for the development of CSC in these cases. Alternatively, it is possible that there is a primary intrinsic scleral disorder which results in shortening or ‘shrinkage’ of the eye. This could explain the scleral features; the increasing hypermetropia; and the axial length shortening in association with CSC and choroidal folds. Indeed, back in 1969 Norton suggested that there may be shrinkage of the sclera leading to distortion of the contour of the eyeball [20]. It is possible that a shortening of the eyeball (inferred by an increasing shortening of the axial length) by whatever mechanism may play a role in the formation of CRF and CSC. None of our patients had an associated inflammatory disorder apart from case 3, who had rheumatoid arthritis. Also, none of our patients showed a resolution of the CRF over time irrespective of the axial length measurements.

An alternative possibility is that in patients with pre-existing hypermetropia, the onset of lens stiffening due to ageing causes excess strain to be exerted through the lens suspensory ligaments as more muscular effort is required to focus as the individual becomes presbyopic. This biomechanical stress could be transmitted through to the posterior pole causing a deleterious tissue response. However, this would not explain why this does not happen in all patients with pre-existing hypermetropia when presbyopia occurs. Finally, there may be an underlying genetic predisposition in these patients for the development of this combination of ocular changes perhaps triggered by another as yet unknown aetiological factor leading to inflammatory changes. However, at present there is no unifying explanation for the aetiology of this disorder. There are drawbacks to our case series, which are explained by this being a case series of patients attending clinics, and not being participants in a study with regular follow up and precise schedules for investigation. For example, we do not have all relevant imaging on all patients, or long term follow up in all seven. In addition, we do not have enhanced depth OCT measurements to examine the role of the choroid in the measurements of the axial length, so cannot be sure that the sclera is the main contribution to the thickening. In addition, the axial length measurements are from non-immersion contact ultrasonography, which is less accurate than immersion biometry/ biometry A scan measurements. One important clinical finding was that despite these changes, visual deterioration in 6 of our 7 cases series was relatively mild. It is also the case that there appears to be a progressive shortening of the eye with shortening of the axial lengths and increasing hypermetropia accompanied by the scleral changes. In this case series we do not have long term follow up on all cases, but 4 of the 7 did show this trend towards ‘shortening of the eyeball,’ which has anecdotally been referred to as ‘shrinking eyeball’. However, the term CHAFES (**C**SC, **H**ypermetropia short **A**xial length, **c**horoidal **F**olds and **E**nlarged/thickened ocular coats, and **S**cleral changes) could be adopted should more cases be reported, as using the term ‘shrinking eyeball’ implies a more sinister outcome and may be unnecessarily alarming. In our case series severe visual impairment occurred in only one patient.

In summary a detailed ophthalmic and systemic evaluation should be performed when CRF are identified, as it is important to exclude a serious systemic condition before the diagnosis of acquired hypermetropia is made. However, once a systemic disorder has been excluded, we recommend initial and regular refraction to identify and address increasing hypermetropia; ultrasonography to characterise any scleral changes, and axial length measurements to make the diagnosis of CHAFES. Recognising that this combination of features can coexist, and may represent a discrete ocular condition, will enable acquisition of further information regarding its natural history, aetiology and pathophysiology. In addition, precise and detailed measurements of the axial length and layers of the eyeball over time will address the question of whether the eyeball is shortening over time.

## Data Availability

All data generated or analysed during this study are included in this published article.
